# Reindeer eyes seasonally adapt to ozone-blue Arctic twilight by tuning a photonic tapetum lucidum

**DOI:** 10.1098/rspb.2022.1002

**Published:** 2022-06-29

**Authors:** Robert A. E. Fosbury, Glen Jeffery

**Affiliations:** ^1^ Institute of Ophthalmology, University College London, 11–43 Bath Street, London EC1V 9EL, UK; ^2^ European Southern Observatory, Karl-Schwarzschild-Straße 2, 85748 Garching bei München, Germany

**Keywords:** tapetum lucidum, seasonal adaptation, structural colour

## Abstract

Reindeer are the only mammal known to seasonally adapt their eyes to the extremely blue colour of the extended twilight that occupies a large part of the winter 24 h cycle in their Arctic habitat. We describe the atmospheric phenomenon that results in this extreme spectral change in light environment. Reflectance spectroscopy is used to characterize the photonic nanostructure that generates the reflective region of the tapetum lucidum behind the retina. A model is proposed to explain the reversible reformatting of the reflector by seasonal changes in the volume of interstitial fluid within the two-dimensional photonic crystal of parallel collagen fibrils. This model is tested by allowing slow evaporation of the fluid from both summer and winter tapetum surfaces while monitoring changes in the reflectance spectrum. Coupled variations in the spacing and the degree of order of the fibril packing can transform the typical gold-turquoise colour of such a tapetal reflector to a deep blue that matches the peak spectral irradiance of twilight. The mechanism we describe might be applied by other animals with similar tapeta that experience prolonged changes in light environment.

## 1. Introduction

Arctic reindeer (*Rangifer tarandus*) are known to exhibit seasonal changes in the spectral reflectance of their tapetum lucidum, a reflecting layer behind the retina which increases the fraction of incoming photons detected in the eye [1]. During the extended exposure to summer daylight, the tapetum reflection shows a gold-turquoise colour similar to many other mammals [2–4]. During the long Arctic winter, with the sun below the horizon, the reflection transforms to a deep blue. This change was assumed to be an adaptive response to the seasonal change in the spectral irradiance of environmental light at high latitudes. It was suggested that the change in intra-ocular pressure caused by long periods of pupil dilation might trigger a change in the fibril spacing of the collagen nanostructure forming the photonic crystal tapetum fibrosum in these animals. The physical process that might achieve this colour change was not explored, nor was the seasonal change in the spectrum of the environmental light related to physical processes in the atmosphere.

Increased visual sensitivity at the blue end of the visible/near-ultraviolet (UV) spectrum is thought to aid the reindeer in foraging and predator detection under the low irradiance of winter twilight [5–7]. This is a consequence of the general decrease in reflectance of many organic materials towards shorter wavelengths which increases their contrast against the high achromatic albedo of snow at visible and UV wavelengths [8–10].

The intensity and spectral quality of environmental skylight on Earth changes with solar altitude and with variations in the physical state of the atmosphere. While the daytime environmental light conditions can vary over an intensity range of five log units, the changes in spectral quality between direct sunlight and overcast in an open landscape are relatively modest [11] although the local environment, such as vegetation cover, can have a strong influence [12]. With the sun more than 18° below the horizon, the remaining spectral irradiance from the sky, originating predominantly from starlight, periodic moonlight and airglow, loses the deep blue of twilight and reverts to a spectral distribution more similar to daylight but up to nine log units fainter [13].

The most significant change in spectral quality in an open landscape occurs during twilight when the solar altitude ranges from just above the horizon to −18°. This radical change in the visible spectrum of the sky irradiance is not very apparent to human vision as the eye adapts to the gradual change and, as twilight progresses towards night, the rod photoreceptors replace cone-mediated colour vision. The physical reason for the extremely blue colour of twilight, which has been known since the early 1950s, is atmospheric absorption in the Chappuis band of ozone [14]. In the daytime, the Chappuis absorption is very weak and has little effect on sky colour, but with sun below a few degrees in altitude, where it grazes the atmosphere tangentially, the optical path is up to 40 times greater than that to the zenith and the colour of the sky is transformed by the removal of much of the yellow/orange irradiance. While it may appear like a continuation of the blue of a clear sky resulting from Rayleigh scattering by air molecules, the twilight ozone blue has an entirely different physical cause and is ever-present, regardless of cloud cover.

**Figure 1.**
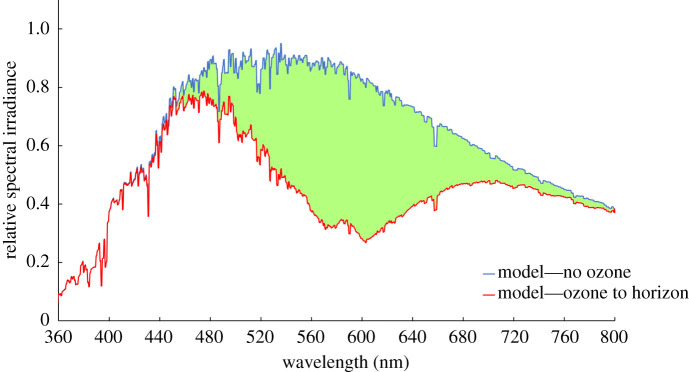
The effect on incoming sunlight of the ozone Chappuis band absorption ‘filter’ on the environmental light during twilight. The ozone column density in this model corresponds approximately to sunset/sunrise and the green-shaded area represents the light removed from the sky, resulting in a predominantly blue environment with a residual component in the far red. This is radically different in spectral shape and physical causation from the daytime blue sky. The twilight sky seen from the ground is bluer that this schematic model since the solar photons illuminating the sky are Rayleigh scattered by the upper atmosphere, a process that favours blue photons over red ones by a factor of approximately 6. (Online version in colour.)

**Figure 2.**
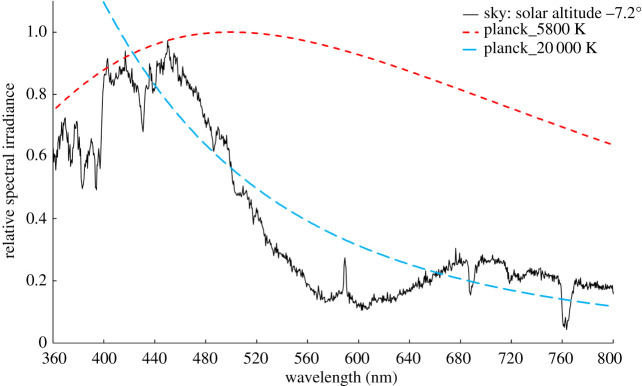
The measured spectral irradiance of the sky at mid-twilight (solar altitude −7.2°). This is close to the maximum effect of ozone absorption on the colour of the downwelling irradiance from the scattering of sunlight by the illuminated atmosphere within and above the ozone layer. The dashed lines show thermal (Planck) spectra with temperatures of 5800 K (approx. sunlight) and 20 000 K. The narrow emission peak at 590 nm results from the resonance scattering of solar photons in the sodium D-lines from atoms coming from ablated meteors that form a layer between 80 and 105 km altitude, above the ozone layer. The strong, broad absorption bands at the red end of the spectrum are from O_2_ and H_2_O in the atmosphere. At high latitudes, the aurora can radiate strong emission lines, principally at 558 and 630 nm, from neutral oxygen atoms above an altitude of around 100 km. (Online version in colour.)

The ozone layer, which occupies altitudes from about 12–40 km, acts as a colour filter spanning the sky. Figure 1, constructed from an atmospheric extinction model familiar to astronomers [15], illustrates the extent of the sunlight spectrum absorbed by ozone when the sun is close to the horizon. This is before the incoming light is directed towards the ground by Rayleigh and aerosol scattering, both of which further tilt the spectrum towards the blue. The blue colour reaches its extreme during mid-twilight when the sunlight travels through the maximum column of ozone and is subsequently Rayleigh scattered from high altitudes before reaching the ground. Both of these processes combine to result in the observed spectrum, shown in figure 2, that was obtained when the solar altitude was −7.2° and can be characterized by a colour temperature around 20 000 K which, with a peak spectral irradiance near 450 nm, is considerably bluer that sunlight.

We calculate that reindeer, when occupying the northern circumpolar regions at a latitude of around +70°, experience 8–11 h of very blue twilight irradiance per day from early September through to April. In a snow-covered landscape, the substantial upwelling irradiance [8,9] retains the dominance of blue light due to the high albedo in the blue and UV. There is evidence that reindeer may exploit the UV content of the winter environment by having a cornea and lens that do not fully block UV, with rod and cone receptors that both respond to it [16].

**Figure 3.**
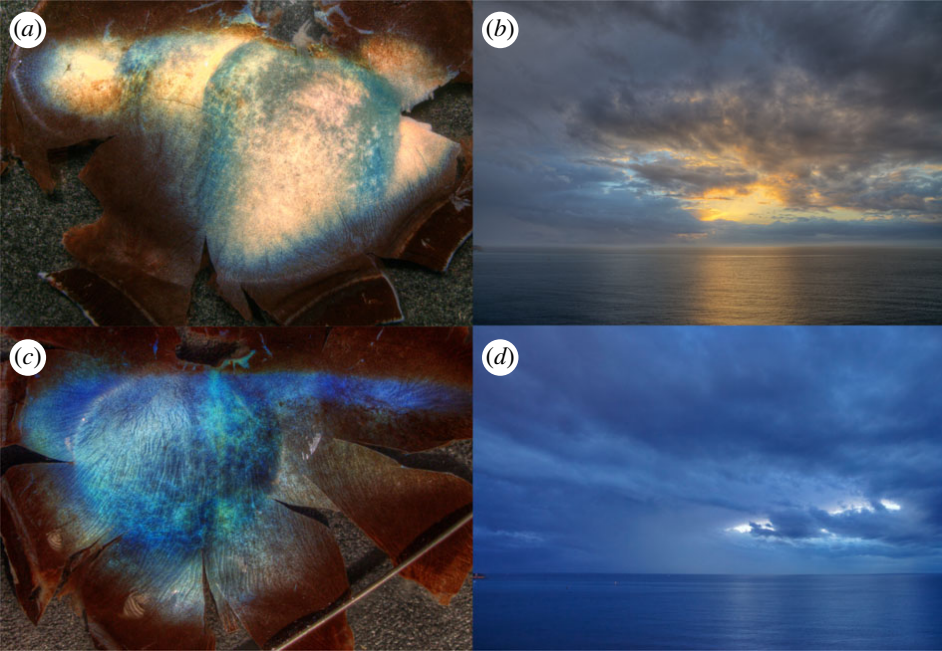
The summer and winter tapetum colour compared with environmental light with the Sun above and below the horizon. The left of the image shows the tapeta taken from reindeer (*a*) in summer (RS3) and (*b*) in winter (RW2). The extended region along the top of these images is referred to as the ‘horizon’, while the vertical extended region offset from the centre is called the ‘area centralis’. On the right are two images of the coastal sea, taken (*b*) with the Sun at an altitude of +2.3° and (*d*) with identical camera white-balance settings, with the Sun at −5.8°, the end of civil twilight. The lower pair of images clearly illustrate the adaptation of the winter tapetum to twilight conditions. © Authors.

Here we present new reflectance measurements of both summer and winter reindeer tapeta that broadly confirm previous reflectance spectra [1]. However, we reduce the diluting specular reflection from the surface layer of the tapetum by making the measurements under water. We also make new separate measurements at points away from the central tapetum and present comparison measurements of roe deer (*Capreolus capreolus*) and European bison (*Bison bonasus*) eyes which have a similar photonic tapetal structure. We show how the winter tapetum in the reindeer is well matched in colour to the twilight spectral irradiance (figure 3), providing maximum visual sensitivity at the shorter wavelengths where the contrast of critical objects in the environment is optimized. A principal new contribution is to propose and explore a two-dimensional photonic crystal model in which the reflectance spectrum can be tuned between its summer and winter states by making a substantial fractional change in the volume of the inter-fibril fluid that determines the collagen rod spacing. This produces a change in the reflectance spectrum that transforms the approximately achromatic summer mirror to a more highly tuned blue peak in the winter. We test this model by allowing both summer and winter tapeta to lose inter-fibril fluid by evaporation while monitoring the reflectance spectrum. It is proposed that such a fluid volume change might be triggered by the known seasonal change in intra-ocular pressure [1] but this possible mechanism is not explored here.

## 2. Material and methods

### (a) Atmospheric models and environmental light exposures

The spectra shown in figure 1 were computed using the methodology and molecular absorption and scattering cross-section data described in [15]. For this illustration, only Rayleigh and aerosol scattering and ozone absorption were included. These three processes alone dominate the effects on colour of the environmental light within the animal's retinal sensitivity range. The ozone column density is chosen to represent the situation when the sun is close to the horizon near the start and end of twilight, defined to be when the solar altitude is between 0° and −18°.

The annual exposure of animals to twilight conditions was calculated for a latitude of +69.5° using the calculator provided by the US NOAA Global Monitoring Laboratory (https://gml.noaa.gov/grad/solcalc/calcdetails.html) which provides a calculator to derive such quantities. Although this procedure uses the strict solar altitude definition of twilight, the effect of ozone absorption on sky colour persists until the altitude reaches up at least to +10°.

### (b) Eye preparation and measurement

Eyes from reindeer and two other ungulates were prepared. Eyes were removed rapidly at the point of death and fixed in 4% paraformaldehyde in phosphate buffer. The reindeer (*Rangifer tarandus*) eyes were collected at a slaughterhouse in northern Norway at a latitude of approximately 70 degrees north and one was measured from each of two winter- and two summer-killed animals. Bison (*Bison bonasus*) eyes were collected from the Zoological Society of London. Measurements were made of one eye in the best condition. Roe deer (*Capreolus capreolus*) eyes were collected following culling in the field in the UK. Measurements were made of three eyes from different animals. Only the reindeer eyes were selected by season. Eyes remained in fixative for extended periods. They were then dissected, removing the anterior structures and the retina. The choroid and the tapetum were then dissected free and washed in distilled water.

The reflective regions of the reindeer tapeta are approximately 60 mm along the horizon and 30 mm vertically across the area centralis. The extracted tapeta were partially cut in a radial direction to allow them to lie approximately flat at the base of a Petri dish and be covered with distilled water. In a similar way, the tapeta of three deer and a bison were prepared and measured for comparison purposes.

The reflectance measurements were carried out with a Maya 2000 Pro spectrometer (200–1100 nm), an HL-2000 tungsten-halogen lamp and a 7-fibre-coupled reflectance probe. Measurements were made with the probe under water to minimize specular reflectance from the tapetum surface and were calibrated with a WS-1 Diffuse Reflectance Standard. The low blue-UV output of the HL-2000 lamp limited the best performance of the system to wavelengths longer than 350 nm but we did perform a limited number of measurements using a DH-Mini deuterium lamp which extended the wavelength coverage to 200 nm. The spectrometer and associated equipment were from Ocean Optics (now Ocean Insight, Duiven, The Netherlands). The reflectance probe integrated over an area of approximately 2 mm^2^, and measurements were spaced over typically 10–15 points covering the reflective region of the tapetum. A spectrum obtained from a non-reflective region of the sample was subtracted from every measurement as a background correction.

We present the reflectance results as relative over the range 0 to 1. Using a reflectance probe under water with a sample that could not be completely flattened made absolute measurements unfeasible. Any pressure applied to the tapetum to flatten it would probably produce local changes in spectral reflectance. The spectral reflectance peaks are broad but have high signal-to-noise and are measured to better than ±3 nm.

### (c) Tapetum structure modelling

Like other ungulates, the reindeer tapetum consists of collagen fibrils suspended in a fluid (predominantly water) and known as a tapetum fibrosum. For visible light, collagen has a refractive index ranging from about 1.50 in the blue to 1.47 in the red [17] and water of 1.34–1.33 [18] over the same range; this implies by Fresnel's law that the fraction of light reflected from a single interface is only around 0.3% or less. To obtain a more useful efficiency, a nanostructure that employs the constructive interference of light can achieve a considerably higher value.

The fibrils are aligned perpendicular to the incoming light in a multi-layered structure consisting of several lamellae, each containing 20 or more layers of parallel fibrils. SEM imaging [1,3] suggests that these parallel fibrils are arrayed into an approximately hexagonal close-packed structure. This constitutes a two-dimensional photonic crystal with the capability of reflecting, with high efficiency, within the visible spectrum by the process generally known as Bragg diffraction. Such ordered nanostructures can be tuned to produce a variety of reflectance properties.

**Figure 4.**
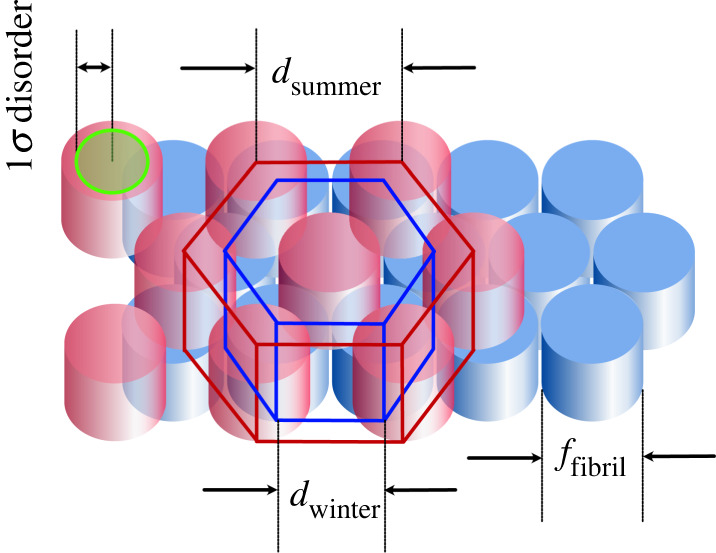
A schematic drawing showing a slice through three layers of collagen fibrils constituting part of the two-dimensional photonic tapetal reflector during summer (red) and winter (blue). The corresponding unit-cells, each containing material from three fibrils, are outlined. The dimensions of the fibril diameter *f* and the Bragg spacing *d* of fibrils in summer and winter are shown. The green circle at the upper left shows the locus of the one sigma variations in fibril position in summer when the fibrils are free to move within the interstitial fluid. It is this disorder in the fibril nearest-neighbour distance that enhances the saddle between the first- and second-order reflection peaks.

A geometrical model of the collagen fibril array was constructed for the purpose of establishing the relationship between the spacings of the fibrils and the resulting reflectance spectrum of the photonic structure. It allows us to suggest and test a possible process for achieving the observed seasonal transformation. The model consists of a hexagonally packed fibril bundle flushed with an inter-fibril fluid assumed to have the refractive index of water. Figure 4 shows a slice perpendicular to the fibrils to illustrate the geometry. A unit-cell of this structure comprises fractions of seven fibrils with a spacing between nearest-neighbour fibril centres of *d*, corresponding to the spacing of diffraction lines in a two-dimensional Bragg crystal. The length *d* can then range between the close-packed state where it equals *f*, the effective diameter of a fibril, and *f* *+* *s* where *s* is the physical spacing between the nearest-neighbour surfaces of the fibrils. We then calculate, as a function of *d*, the volume *V* of the inter-fibril fluid in a unit length (along the fibril axis) of a unit-cell of the structure.

The volume *V* available for the inter-fibril fluid in a unit-cell of length one is the volume of the entire hexagonal unit-cell minus the volume of the cell occupied by collagen fibril which is

$$V=\frac{3\sqrt{3}d^{2}}{2}-\frac{3\pi f^{2}}{4}$$

This gives us a relationship between *d*, which determines the wavelengths of the of the reflectance peaks, and the fluid volume *V* for a given fibril diameter *f*.

To validate this model as a process that can explain the tapetum reflectivity changes, we used slow evaporation of the inter-fibril fluid while monitoring the reflectance spectrum. This was achieved by placing a wet region of tapetum approximately 5 by 10 mm on wet filter paper beneath a vertical reflectance probe. In this case, surface specular reflections were avoided by angling the sample 20° from horizontal. Reflectance spectra were then obtained approximately every 5 min over a period of 2 h until the tapetum was dry, when the reflectance becomes very low. Measurements of the two summer and the two winter reindeer eyes were carried out under constant temperature (24°C) and humidity (RH 45%).

### (d) Husky fur reflectance

Reflectance measurements were made of fur cut from two live husky dogs in winter. The samples were taken from the neck (dorsal), shoulder and flank. These were measured with the reflectance probe using the HL-2000 lamp and calibrated with the WS-1 standard. As the spectral shape was similar for all samples, there were averaged to produce a representative spectrum.

## 3. Results

**Figure 5.**
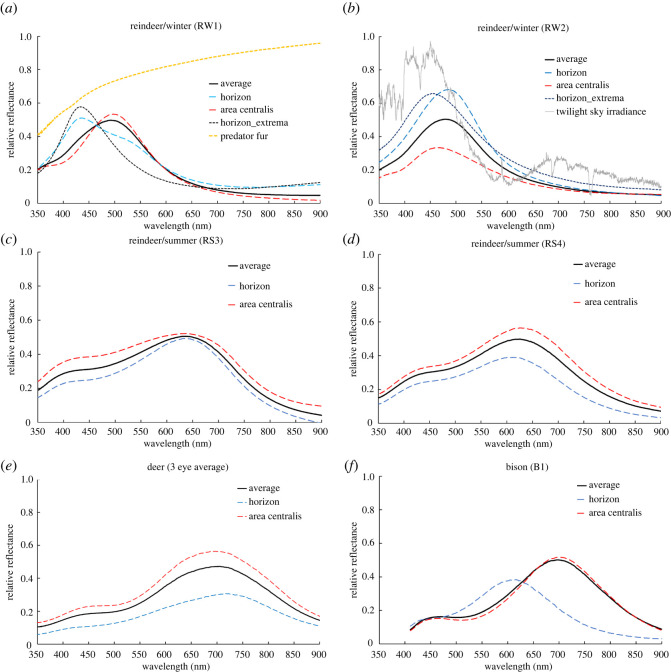
Tapetum spectral reflectance measurements of the reindeer (*a*,*b*) in winter and (*c*,*d*) in summer, (*e*) the deer (an average of the three eyes measured) and (*f*) the bison. Each plot shows the average of the spectra from all measured points over the tapetum (thick black line) and separate averages of the points along the horizon (dashed blue) and within the area centralis (dashed red). For the winter reindeer, there is the additional curve showing the reflectance of the shortest wavelength measured peak along the horizon (dashed black); (*a*) includes (dashed orange) the reflectance spectrum of the fur of the wolf (husky) while (*b*) reproduces the twilight spectral irradiance from figure 2.

The sequence of plots in figure 5 shows the reflectance spectra of the two winter and the two summer reindeer tapeta, an average of the three deer and the single bison. Each plot shows the average of all the position measurements and, as dashed lines, those from the horizon and area-centrals regions. For the two winter reindeer tapeta, an additional spectrum shows the most extreme blue reflection from the tapetal horizon.

In figure 5*a*,*b*, the winter reindeer show a single reflectance peak in the blue that we interpret as the first-order Bragg reflection. The remaining measurements all show a prominent red peak at 600–700 nm with a secondary peak or shoulder in the blue. In wavelength units, all of these red peaks are broader than those of the winter reindeer by approximately one-third.

To understand the nature of the seasonal reflectance transition, we assume that it is due to a change in the fibril centre-to-centre spacing *d*, which determines the wavelength of the first and subsequent orders of the Bragg reflectance. This could be the result of either a change in the fibril diameter *f*, or an increase in the spacing between the fibril surfaces *s* keeping *f* constant. In order to distinguish between these two possibilities, we have analysed spectra taken during the slow evaporative drying of tapetum samples and interpreted them using our geometrical unit-cell model.

From measurements of the variation of the peak reflecting wavelength with time, we use the Bragg equation to infer the changes in the fibril spacing, *d*. If the inter-fibril fluid in a summer tapetum decreases in volume, *d* will decrease until the fibrils come into contact and then stall. If the fibrils themselves change diameter the reduction in fluid might just indicate a gradual desiccation of the collagen and fluid with no clear stalling event.

**Figure 6.**
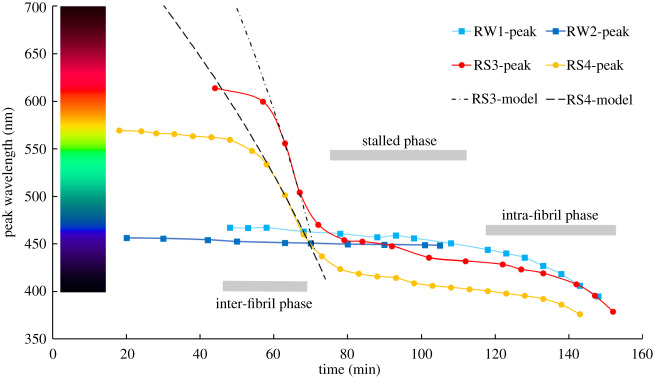
The results of a slow evaporation experiment designed to investigate, by spectral reflectance, the collagen fibril configuration change between their summer and winter packing within the tapetum. This shows the behaviour of two summer (red and orange curves) and two winter (dark and light blue) tapeta measured during the process of drying over a period of about 2 h. The dashed black lines show models of the peak wavelength change computed from the unit-cell during the period when the inter-fibril fluid is evaporating at a constant rate. (Online version in colour.)

Figure 6 shows the results of these four evaporation experiments. The two summer samples commence with a slow decrease in the first-order peak wavelength with time, likely to result from the initial evaporation of excess surface water covering the tapetum surface. The summer tapeta then undergo a phase of rapid peak wavelength decrease until stalling at a wavelength close to that of the winter tapeta peaks. The two winter tapeta show just a very slow wavelength decrease extending beyond the stalling of the summer samples. The subsequent time evolution of both summer and winter tapeta are similar with the final downward slope probably arising from the desiccation of the fibrils themselves. These data suggest that the measured winter/summer change in *d* is the result of a change in inter-fibril fluid volume rather than in fibril diameter *f*.

The first-order peak reflectance wavelengths for the four reindeer eyes are used to estimate the dimensions of the unit-cell in our model of the fibril. Using the evaporation curves shown in figure 6, we derive the change in the volume of the inter-fibril fluid between the summer and winter tapetum configurations. The peak wavelengths give directly, via the Bragg equation using the refractive index of water, the fibril centre-to-centre spacing *d* shown as the first column in table 1.

**Table 1.** The collagen fibril model unit-cell parameters derived from the measured wavelengths of the first-order peaks in the winter (RW1 and RW2) and summer (RS3 and RS4) reindeer tapeta. The *measured* peak wavelengths are from spatially averaged reflectance spectra of the four reindeer tapeta, and the linear and volume dimensions are *derived* from our geometric model of the unit-cell using the Bragg equation. The second column shows the peak wavelengths while the following three columns show the derived values of the unit-cell dimensions *d* and *f* defined in figure 4; *s* is included here since its values are discussed in [1]. The final three columns show, respectively, the volume of the entire unit-cell containing fibril and fluid, the volume of the fluid only and, finally, the volume of fluid remaining in the summer samples when the wavelength stalls after the evaporation of the inter-fibril fluid as shown in figure 6. Note that the unit-cell volume changes by a factor of approximately 2 between winter and summer while the fluid content changes by approximately a factor of 10.

Eye	*λ_*peak*_* (nm)	*f* (nm)	*d* (nm)	*s* (nm)	*V*_unit-cell_ (nm^3^)	*V*_fluid_ (nm^3^)	*V*_fluid-stall_ (nm^3^)
RW1	482	180	~*f*	~0	8.42 × 10^+4^	7.8 × 10^+3^	—
RW2	466	174	~*f*	~0	7.87 × 10^+4^	7.3 × 10^+3^	—
RS3	630	174	235	61	1.43 × 10^+5^	7.2 × 10^+4^	7.3 × 10^+3^
RS4	625	162	233	71	1.41 × 10^+5^	7.9 × 10^+4^	6.4 × 10^+3^

From the stalling behaviour seen in the summer evaporation curves, we make the assumption that the values of *d* for the winter tapeta (RW1 and RW2) are very close to the fibril diameter *f*. This implies that, in the winter state, the volume of the inter-fibril fluid is a minimum with the fibrils essentially in contact with hexagonal packing. The values for *f* and *s* shown in table 1 for the winter tapeta are simply *f ~ d* and *s ∼ 0*. Note that the values of *s* in winter and summer tapeta were estimated from SEM images in [1] to be 26 and 43 nm, respectively, although these are likely to be influenced during sample preparation in a manner that is avoided with our wavelength measurements.

For the summer tapeta (RS3 and RS4), the values of *f* and *s* are obtained from the model fits to the steeply falling regions of the evaporation curves. The two model fits in figure 6 assume that the evaporation rate in the inter-fibril phase is constant in time. The final three columns in table 1 show the derived unit-cell volumes *V* for the four tapeta: *V*_unit-cell_, the full (fibril + fluid) unit-cell volume: *V*_fluid_, the fluid volume in the tapetum as measured; and *V*_fluid-stall_, the fluid volume for the close-packed fibrils.

**Figure 7.**
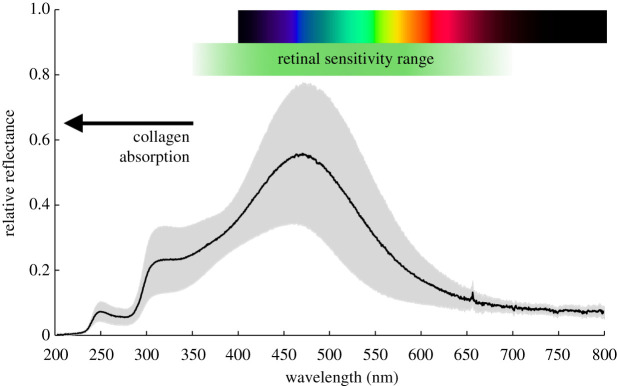
The far-UV/visible reflectance spectrum of one of the winter reindeer tapeta averaged from five positions within the tapetum. The grey regions indicate one standard deviation of the sample measurements. Towards the UV, the presence of significant collagen absorption decreases the overall reflectance of the tapetum as well as reducing the transmission of other collagen structures in the eye. In this spectrum, the peak second-order Bragg reflection is absorbed but a first second-order saddle remains to extend the tapetum reflectance down to approximately 350 nm where the retina retains sensitivity. The green bar shows the approximate range of retinal sensitivity. (Online version in colour.)

Figure 7 shows an average of five reflectance measurements of one of the winter tapeta (RW1) made with the UV deuterium lamp from positions spread over the tapetum surface with the grey regions indicating one standard deviation of the sample. The green bar indicates the approximate range of the animal's retinal sensitivity. The spectrum shows a clear signature of collagen absorption increasing below 350 nm and reaching an absorbance peak at 270 nm [19].

To address the effectiveness of the blue enhancement of the winter tapetal reflectance, we used reflectance spectra of husky dogs as representative of the reindeer's primary predator, the wolf (figure 5*a*). This shows a decrease in reflectance of a factor of at least 20% between the wavelength of peak summer visibility around 550 nm and the winter peak of tapetum horizon reflectance of around 450 nm. This is intended as representative of the wolf as the principal reindeer predator, recognizing that a decrease in reflectivity of fur towards the blue and UV is typical of other potential predators.

## 4. Discussion

Many animals that are active under low-light conditions have evolved a method of increasing their visual sensitivity by developing a layer directly behind the retina that acts as a mirror, a scatterer or, perhaps in some cases, a fluorescent screen [20] to redirect incoming light back through the photoreceptors for a second pass. This increases visual sensitivity but at the cost of some visual acuity. A number of different physical solutions have evolved to provide this capability over a wide range of animal species [4]. A remarkable set of coloured drawings of animal tapeta was published by Johnson in 1901 and has since been reproduced in higher quality [2].

An ideal photonic nanostructure can provide a high reflectivity even when constructed from materials that have only a small difference in refractive index, such as collagen and water. When the structure is highly ordered, however, the reflected light comprises a series of narrow spectral peaks decreasing in strength towards shorter wavelength. In the tapetum fibrosum, the bandwidth of the reflected peaks is broadened by the degree of disorder in the spacing of the collagen fibrils about a median value that determines the wavelengths of the peaks.

In the model that we have proposed, the magnitude of this disorder is high in the summer tapetum as a consequence of the freedom of the fibrils to occupy random displacements within a large volume of inter-fibril fluid (figure 4). By placing the first-order reflectance peak at the red end of the spectrum, at or beyond the range of the animal's vision, the first- and second-order broadened reflectance peaks merge to form a saddle that extends over the entire range of the retinal sensitivity to form an almost achromatic mirror. This is especially clearly illustrated in the modelling shown in fig. 18a of [21].

A reduction in the volume of the inter-fibril fluid in the winter tapetum results in both a shift towards the blue of the reflectance peaks and an increasing degree of order in the fibril spacing. The latter is due to tighter positional constraints imposed by the approach to the closest possible packing where the fibrils are essentially in contact in a hexagonal pattern. The increase in regularity of fibril spacing narrows the bandwidth in wavelength but retains a saddle that bridges the first and second orders [21]. While the first-order peak in winter matches the spectral distribution of twilight, the extension of the saddle towards the second-order peak in the UV maintains the efficacy of the tapetum down to a wavelength of around 350 nm where collagen begins to absorb but where the reindeer retina retains good sensitivity [16]. This broad spectral range of blue-UV vision potentially increases the contrast advantage.

**Figure 8.**
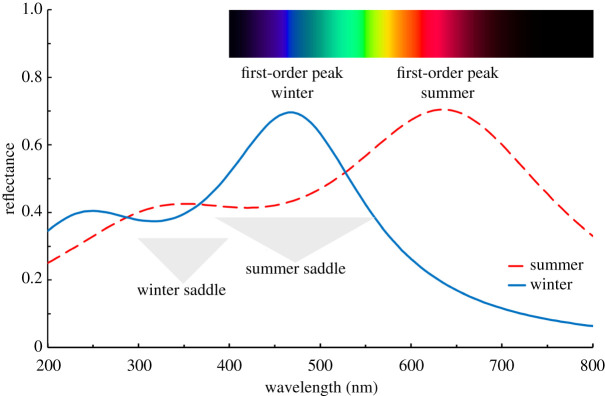
A schematic illustration of the transformation between the summer and winter tapetum reflectance spectra. We propose that this happens by a decrease of the spacing of the parallel rod-shaped fibrils in the crystal when the volume of the inter-fibril fluid decreases by an amount that allows them to approach a close-packed hexagonal structure. When the fibrils float within a significant volume of the liquid they can lose the regularity of this close-packed structure and introduce a significant degree of disorder into the crystal that broadens the first and higher order spectral peaks to produce a ‘saddle’ of reflectance stretching between them. In the summer, this saddle encompasses the visual sensitivity range of the animal, providing it with almost achromatic mirror surface which ranges in appearance as seen by us, from pale gold to turquoise. Note also the contributions of the second-order reflections to UV reflectivity around 350 nm in both summer and winter configurations [16]. (Online version in colour.)

The schematic drawing in figure 8, which neglects the UV collagen absorption seen in figure 7, illustrates the effects we have discussed and, in particular, shows the importance of the saddle region between the orders that provides reflectance over the range of the animal's cone vision in the summer and the extended UV capability in the winter. It can also be appreciated that small variations on the shape of the saddle region can explain the variations between the gold and turquoise appearance of many photonic tapeta.

The final three columns in table 1 show that, to achieve this wavelength shift and reordering, there is a change of a factor of approximately 10 in the volume of the inter-fibril fluid in a unit-cell. We also see that the change in total volume (fluid + fibril) of the unit-cell between seasons is near a factor of two. How can such changes be accommodated within the tapetum?

As we were not able to measure the same tapetum in both summer and winter, we cannot be sure how much the reflective tapetal area changes with season. However, from examining all the summer and winter tapeta, it is clear that any difference is small. Assuming that the length and number of the fibrils does not change, this volume change must occur as a result of the change in area of the unit-cell viewed perpendicular to the fibrils (figure 4). This means a factor of about approximately √2 in the thickness, and a factor of approximately √2 in one dimension in the plane, of the tapetum. Thus, for the winter configuration, the number of fibril layers within a lamella must reduce by approximately 40% to provide the extra unit-cells to cover the same tapetal area. This will entail some re-packing of fibrils within a lamella but we see no reason why this should not happen spontaneously as fluid is removed. To preserve the tapetal area, all this change must be accommodated as a decrease in its thickness by a factor of approximately 2. The total thickness of an ungulate tapetum is between 100 and 200 µm [4] and, for a reindeer to accomplish this reduction in winter, it could probably be achieved without undue disturbance of blood supply and nerve connections that perpendicularly penetrate the tapetum. This may result in a small focus change but, under low-light conditions, the animal will rely more on contrast and movement than on acuity to identify a predator.

While all this has been learned by examining eyes from animals that live in Arctic regions, we have yet to understand how the seasonal structural change in the eye is triggered and the biochemical pathways that bring it about. It is known that winter animals have a significantly increased intra-ocular pressure, probably as a result of continuing pupil dilation in low-light conditions blocking ocular drainage. While this increased pressure may not directly squeeze the collagen fibrils to a closer spacing, it may change the fluid balance within the eye and drain the tapetum in the way that we have described. However, the change is triggered, it clearly must be reversible.

Such an application of a photonic structure in an animal is in marked contrast to those of animals that employ structural coloration to create brilliant, often iridescent markings for the purpose of high visibility. The introduction of disorder into a nanostructure that has a characteristic scatterer spacing allows a marked reduction of iridescence and a broadening of the spectral bandwidth of a reflecting surface. In the eye, this enables an existing collagen structure behind the retina to be shaped into a constructively interfering mirror that enables a substantial increase in visual sensitivity by increasing the effective path length of photons among the retinal photoreceptors. Although we have not attempted to measure the absolute reflectance of the tapeta in our sample, it is likely, based on the large number of the constituent fibril layers, to range from 50% to approaching 100% close to the first-order reflection peak. This is especially pertinent to the winter tapetum where the peak lies within the range of the reindeer cone photoreceptors and the hexagonal structure is most highly ordered. It is likely therefore that the blue tapetal reflection is a significant contributor to the substantially increased electroretinogram responses from winter reindeer measurements relative to those in the summer [1].

A second type of disorder is seen in the bovine tapetum described in [3], which shows that the depth of the tapetum contains several lamellae, each containing multiple layers of parallel fibrils. Each consecutive lamella is rotated in the tapetum plane by an angle that breaks the azimuthal symmetry and leads to a more isotropic reflection. From the SEM images, it appears that the angle of rotation might be a few to 10 or more degrees between adjacent lamellae [3] which would contribute to azimuthal uniformity of the tapetal reflection.

While the reindeer is the only mammal known to undergo these seasonal changes, the nature of the tapetum fibrosum might allow such a change to occur in other animals using this tapetum type that are subject to long periods of twilight or other sustained periods of environmental light with an extreme colour.

While most reindeer predators have fur that decreases in reflectivity in the blue-UV, recent work has shown that the polar bear may maintain its white appearance by using another photonic capability, namely fibre optic hair [22,23], to successfully hunt reindeer in a terrestrial environment.

We have not had access to eyes obtained from animals after the autumnal or before the spring equinoxes and so have no measurements relating to the transition between the summer and winter tapetal configurations. If, as is likely, the transition occurs slowly and reasonably uniformly as the light fades and re-appears, it is possible that the tapetum colour might be observed to exhibit the spectral colour sequence from gold-yellow-green-blue as the first-order reflection peak moves from the far red to deep blue. This might be observed in live animals as changes in eye-shine colour. The sequence would reverse before the spring equinox.

## Data Availability

The data used to plot figure 5 are available as electronic supplementary material, spreadsheet files [24].
